# MAINE REACHING FOR THE SUMMIT: ADDRESSING ALZHEIMER’S DISEASE AND RELATED DEMENTIAS

**DOI:** 10.1093/geroni/igae098.1226

**Published:** 2024-12-31

**Authors:** John Holland, Alfred May, Jr, Eric Frohmberg, Dawn Gordon

**Affiliations:** Maine Center for Disease Control and Prevention, Augusta, Maine, United States; State of Maine Department of Health And Human Services, Maine Center for Disease Control and Prevention, Machias, Maine, United States; Chronic Disease Prevention and Control Programs, Maine CDC, Augusta, Maine, United States; Maine Center for Disease Control and Prevention, Augusta, Maine, United States

## Abstract

Maine is one of the most rural states in the country with 61.4% of its population living in rural areas and thirteen of sixteen counties categorized as rural. Maine is also the oldest state in the country based on a median age of 44.8 years. Maine’s BOLD Program strategized to treat the whole state as rural and determine various means to build the infrastructure for dementia education and services. One of these strategies is to develop a working relationship with primary care through our federally qualified health centers, who serve our target low-income population and are located in our most rural areas of the state. Because federally qualified health centers are established to provide primary care services as well as oral and behavioral health, they are open to partnerships with the Area Agencies on Aging for providing specialized umbrella services to patients with Alzheimer’s disease and related dementias (ADRD) and respite services to families and caregivers of patients with ADRD. This approach builds on the Healthy Brain Initiative Road Map strategies as well as working toward clinical to community best practices in preventing ADRD. A key resource for our work with the lead federally qualified health center has been the New York University Medical School, one of the Public Health Centers of Excellence. This collaboration has resulted in the creation of a dialogue type webinar that utilizes research to practice for early detection, diagnosis, and care planning.

